# Breakage in Two Points of a Short and Undersized “Affixus” Cephalomedullary Nail in a Very Active Elderly Female: A Case Report and Review of the Literature

**DOI:** 10.1155/2018/9580190

**Published:** 2018-09-13

**Authors:** Giuseppe Rollo, Giuseppe Rinonapoli, Paolo Pichierri, Michele Bisaccia, Auro Caraffa, Luigi Meccariello

**Affiliations:** ^1^Department of Orthopedics and Traumatology, Vito Fazzi Hospital, Lecce, Italy; ^2^Division of Orthopedics and Trauma Surgery, University of Perugia, “S. Maria della Misericordia” Hospital, Perugia, Italy

## Abstract

**Introduction:**

Trochanteric fractures of the femur are common in elderly individuals with osteoporosis. The use of cephalomedullary nails is increasing, and they are now the most commonly used fixation devices, especially for the treatment of unstable trochanteric fractures. The nail breakage is not the most common complication of intramedullary nailing. Many scientific papers report nail breakage in a specific location: through the lag screw hole, the nail shaft, or the distal locking hole.

**Materials and Methods:**

We present a case of an 84-year-old patient treated with modular revision hip arthroplasty due to the breakage in two points of a cephalomedullary nail implanted 3 years earlier for a subtrochanteric fracture.

**Results:**

After modular revision hip arthroplasty, the functional results and quality of life have been excellent.

**Conclusions:**

As far as we could determine, this appears to be the first case of a breakage of a cephalomedullary nail in two points after nonunion in a very active elderly female.

## 1. Introduction

Trochanteric fractures of the femur are common in elderly individuals with osteoporosis and are usually treated surgically to facilitate early rehabilitation [1, 2]. Many devices have been developed to fix these fractures, mainly a sliding hip screw (SHS) or a cephalomedullary nail. In terms of load shearing, the cephalomedullary nail is biomechanically advantageous because of its shorter lever arm [3, 4]. The use of intramedullary nails is increasing, and they are now the most commonly used fixation devices, especially for the treatment of unstable trochanteric fractures [5, 6]. Anglen and Weinstein [6] found that from 1999 to 2006, for fixation of intertrochanteric hip fractures, there was a dramatic increase in the preference for the use of intramedullary nails that interlock proximally into the femoral head, in comparison with the use of a sliding compression screw. The intramedullary nail fixation rate increased from 3% in 1999 to 67% in 2006.

The AFFIXUS Hip Fracture Nail System, with its two variants, short and long, is intended to treat stable and unstable proximal femoral fractures, nonunions, or malunions, either pertrochanteric, intertrochanteric, high subtrochanteric, or their combinations, with extended use in case of bone loss due to tumor resections, ipsilateral fractures of the proximal femur and its shaft, impending pathological fractures, etc. Nonunion of trochanteric fractures is relatively uncommon, with a reported incidence of 1–5% [7]. In order to decrease the incidence of failure, several variations of intramedullary nails have been devised [7]. Nevertheless, the newer nail designs and materials can still result in complications such as cut-out of the implant, fracture of the femoral shaft distal to the tip of the implant, or medial migration of the implant [7].

The 1-year mortality after hip fracture can be as high as 20–30% [8]. We present a rare case of a two-point breakage of an Affixus® (Zimmer Biomet™, Warsaw, Indiana, USA) nail due to fatigue of an undersized-diameter and a relatively short-length nail used to fix an unstable trochanteric fracture in a very active elderly female. We also review the literature and discuss the incidence, causes, and treatment of implant failure.

## 2. Case Report

**Figure 1:**
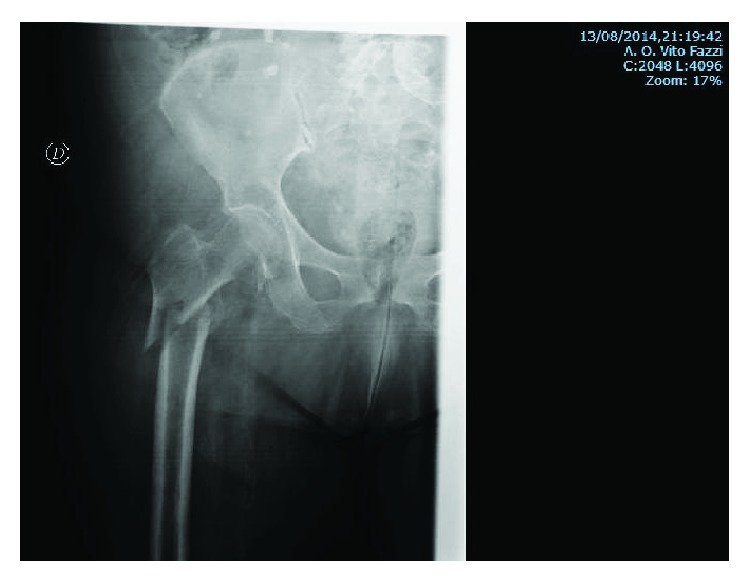
Subtrochanteric fracture with displacement with lesser trochanteric fracture of the femur. According to the AO classification: 31-A3.

**Figure 2:**
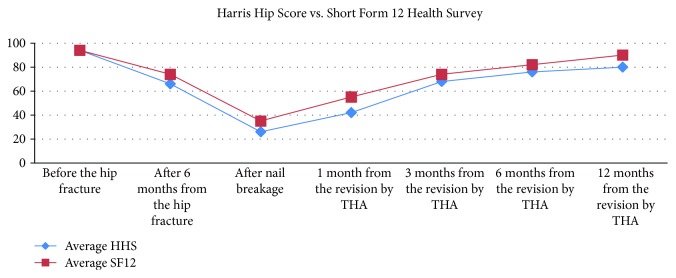
Trend of the patient's HHS and SF-12 before the proximal femoral fracture, through the Affixus® nail (Zimmer Biomet™, Warsaw, Indiana, USA) breakage, and after the definitive THA implantation.

**Figure 3:**
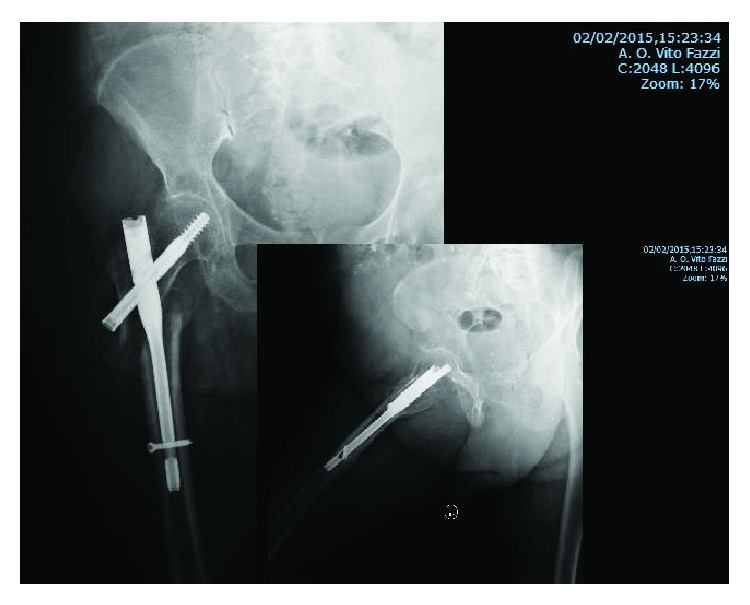
X-rays at 6 months of follow-up. Displacement of the fracture and aseptic nonunion.

**Figure 4:**
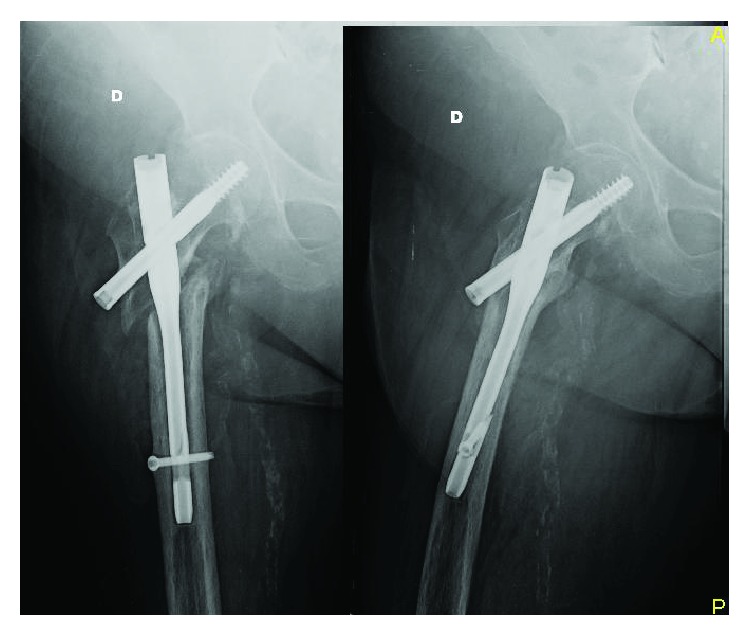
X-rays done in other hospital after 1 year of follow-up showed that the first incomplete breakage was on the hole for the distal static screw. The orthopaedic surgeon did not talk about nail dynamization or nail remotion and revision to the patient.

**Figure 5:**
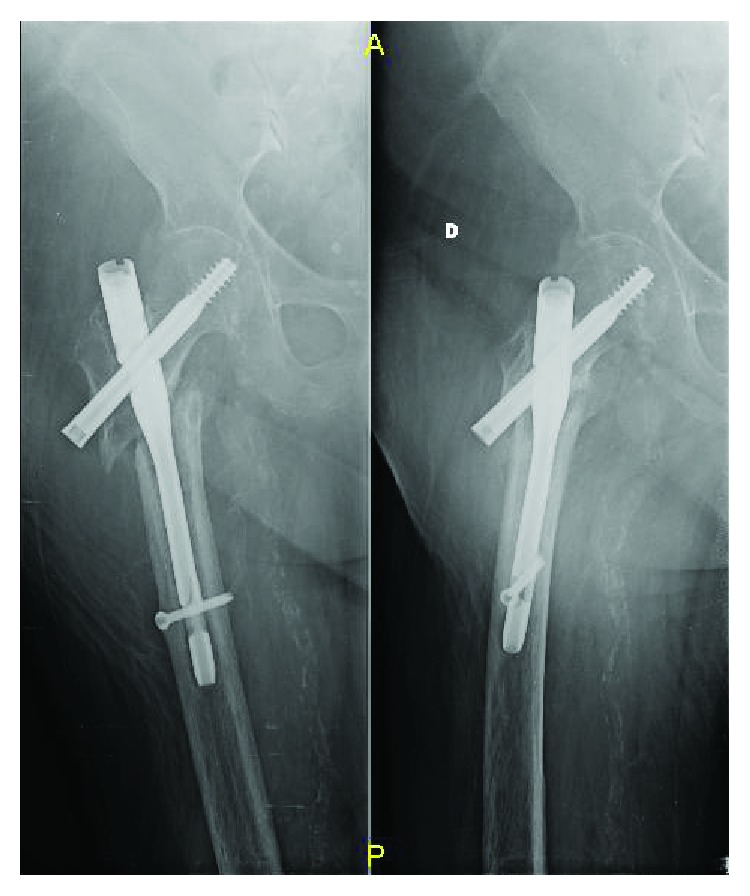
X-rays done in other hospital after 2 years of follow-up showed that the second incomplete breakage was through the barrel for the lag screw while the first incomplete breakage was on the hole for the distal static screw. The orthopaedic surgeon did still not talk about nail dynamization or nail remotion and revision to the patient.

**Figure 6:**
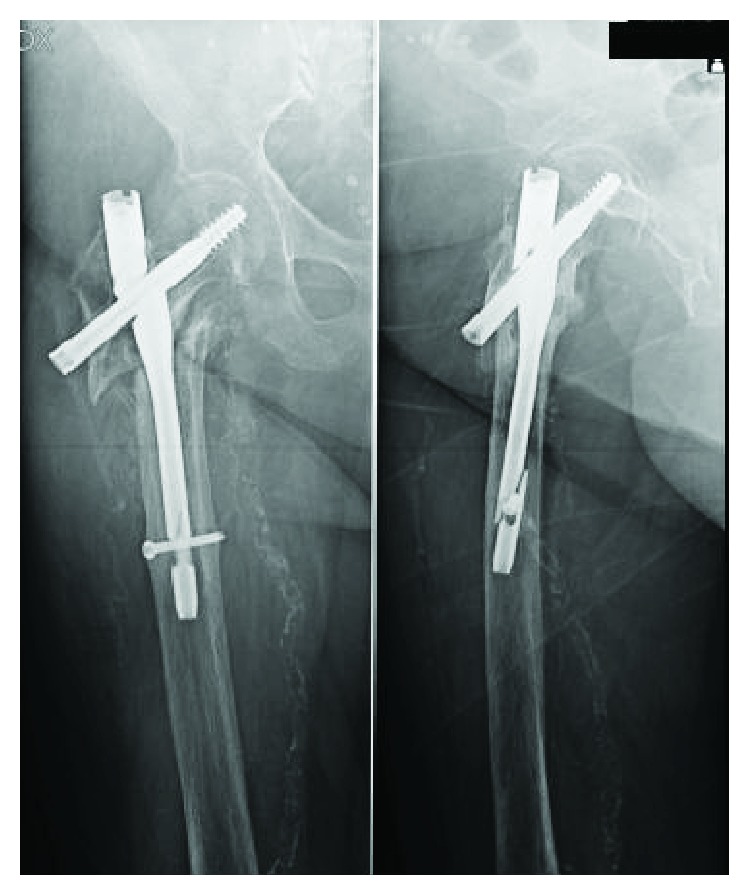
After 3 years, the X-rays (done in Emergency Room) showed the breakage of the nail in its proximal part and distal part.

**Figure 7:**
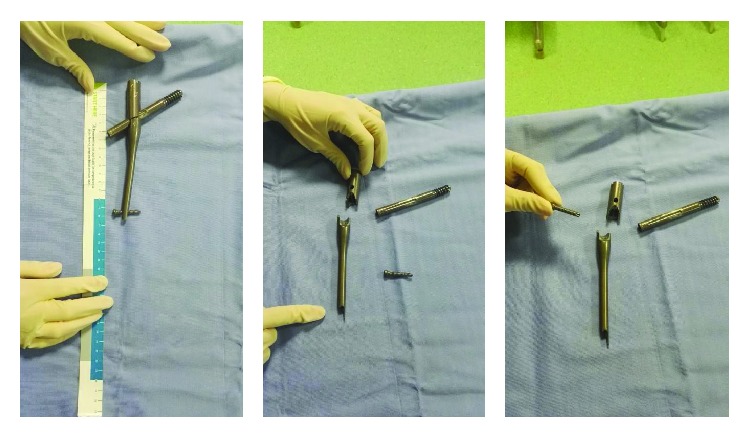
Pictures of the broken nail.

**Figure 8:**
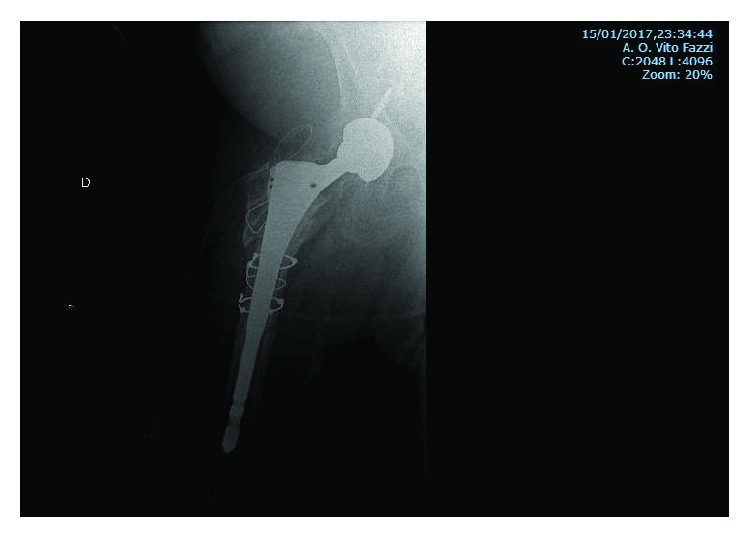
Postsurgery X-ray, after nail removal and replacement with revision THA with two screws to fix the cup and three metal cable cerclages to prevent the breakage during the implant of the stem.

**Figure 9:**
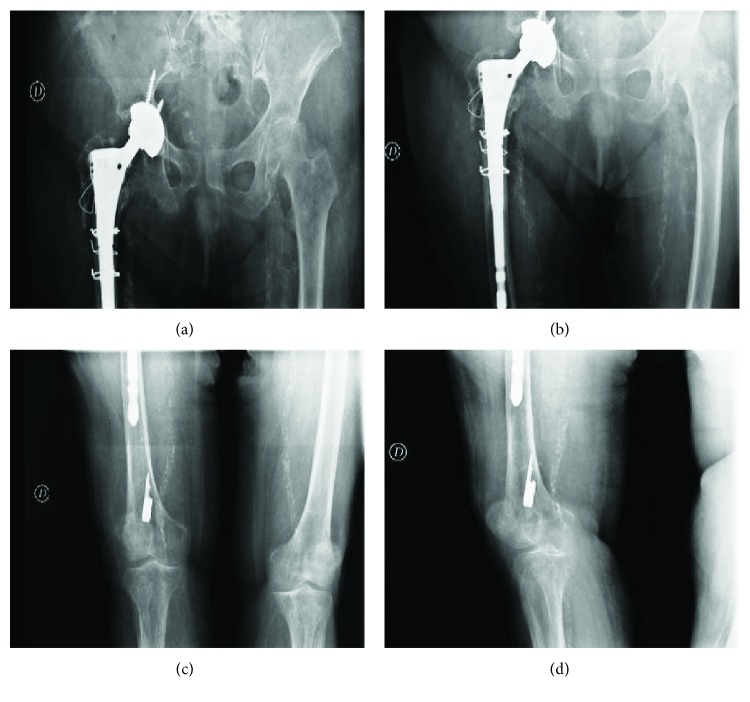
X-rays at 1-year follow-up after surgery. Image (a) shows the integration of the prosthetic cup into the acetabulum. Image (b) shows the bone reabsorption around the stem where there was nonunion. Image (c) shows the absence of stress shielding along the stem and at the stem's apex and the absence of the tip effect. Image (d) shows the apex of the broken nail deposited in the distal metaphysis of the femur.

An 83-year-old woman, with a height of 1 meter and 65 cm and a weight of 85 kg (BMI = 31.22), was transferred to our department because of a reverse pertrochanteric-subtrochanteric fracture AO 31-A3 (Figure 1). The patient had a cardiovascular disease of moderate severity, though her social life was very active, and the Harris Hip Score (HHS) [9] and the Short Form 12 Health Survey (SF-12) [9] were both 94 points (Figure 2). The anesthetic risk was ASA 3 [8], and she did not require intensive care after surgery. After reduction, internal fixation was done using a short Affixus® nail (Zimmer Biomet™, Warsaw, Indiana, USA) which is 180 mm long. The shaft was 9 mm wide, the lag screws were 100 mm long, and one distal static locking screw was used (Figure 3). In the 1st postoperative day, rehabilitation began, and by the 2nd day, she was walking with total progressive weight-bearing. She was discharged on the 7th postoperative day. Six months after surgery, at the last control, the HHS was 66 and the SF-12 was 74, and the radiographs showed subtrochanteric nonunion and medial displacement of the distal fragment (Figure 3). Thereafter, the patient seek medical assistance in another hospital. Six months later, she underwent radiographic studies which showed incomplete breakage of the nail at the hole for the locking screw (Figure 4), though no surgical treatment was indicated. There was no pain in the hip, and 2 years following primary surgery, radiographs done in the other hospital showed further incomplete nail breakage at the hole for the lag screw (Figure 5). No further treatment was planned, and later on, the patient reported mild pain while flexing the hip. One year later, i.e., three years after surgery, the patient seek further assistance because of the sudden severe hip pain, and the radiographs showed complete fracture of the nail at both the proximal and the distal holes (Figure 6). The HHS was 26 points and the SF-12 was 35 (see Figure 2). She underwent revision surgery, with removal of the broken nail (Figure 7) and total hip arthroplasty with a 46 mmØ Plasmacup® acetabular cup with a 28 mmØ bearing liner in polyethylene (Aesculap, B. Braun, Melsugen, Assia, Germany) fixed with two screws of 24 and 32 mm length, a 28 mmØ Prevision® metal femoral head, and a 240 mm long modular revision stem (Aesculap, B. Braun, Melsugen, Assia, Germany), with proximal segment P1/0 mm and distal segment 12 mmØ, with the addition of three free metal cable cerclages (Figure 8). Twelve months after the revision surgery, the HHS was 80 points and the SF-12 was 90 points (Figures 2 and 9).

## 3. Discussion

In this article, a comparison of breakage of the Affixus cephalomedullary nail was made with the Gamma nail (GN) (Stryker™, Kalamazoo, Michigan, USA) and other similar devices (Table 1). The GN is one of the most commonly used devices for the treatment of trochanteric fractures, especially unstable fractures [6, 10–12]. Implant failure of the GN is rare probably because of the material strength and mechanical advantages [13, 14]. In 2013, Iwakura et al. presented a case of breakage of a third-generation Gamma nail used to treat an unstable trochanteric fracture, which was thought to be mainly due to insufficient reduction of the fracture, leading to nonunion and secondary nail breakage [10]. The most common cause of nail breakage is metal fatigue secondary to delayed union or nonunion [3, 8]. The GN was conceived as a temporary implant, subjected to repetitive stress loads, and consequently with limited life expectancy and consequently when there is delayed union or nonunion, metal fatigue can be expected [8]. Iwakura et al. performed a meta-analysis of Gamma nail breakage with a reported range incidence between 0.2% and 5.7% [10]. According to Norris et al., the overall reported incidence of secondary fracture around the nail was 1.7%. The incidence of nail failure has been reduced with the development of the Gamma3 cephalomedullary nail (1.7% versus 2.6%, *p* = 0.03), and it was also found that long nails have a slight tendency towards a lower risk of fracture, although the difference was not statistically significant (1.1% versus 1.7%, *p* = 0.28) [12]. In other studies, there was breakage solely in first- and second-generation Gamma nails, either short or long [13–20]. Another device used in pertrochanteric fractures is the Proximal Femoral Nail® (PFN) (DePuy Synthes, Raynham, Massachusetts, USA). Rappold et al. used PFNs in 61 patients with subtrochanteric fractures, and they had 2 cases of nail breakage at the hole for the lag screw [21]. In 100 pertrochanteric fractures, Rappold et al. used the InterTAN® cephalomedullary nail (Smith & Nephew™, Memphis, Tennessee, USA) for internal fixation of pertrochanteric fractures and found several postoperative mechanical complications including significant collapse of the femoral neck in six (6%) patients, fractures distal to the implant (short nail only, *n* = 75) in six (6%), cut-out in one (1%), infections in four (4%), and implant breakage in one (1%). The implant breakage occurred at the hole for the lag screw 6 months following surgery [21]. Zhao et al. reported on 164 intertrochanteric fractures treated with a Trigen short reconstruction trochanteric anterograde nail and found a 17.0% fracture rate at the tip of the nail, a 15.9% poor reduction rate, and a 41.4% cracking rate of the greater trochanter in type 31-A3 fractures, with two shaft fractures requiring revision. No screw breakage but one cut-out occurred. When the patient underwent revision with total hip arthroplasty. All other fractures healed [22]. The risk of distal shaft fractures was not associated with the patient age, gender, fracture type, or cortical bone index [13–23]. Maniscalco et al. presented a rare case of 31-A2 nonunion and breakage of an EndoViS® nail (Citieffe™, Calderara di Reno, Bologna, Italy), due to failure at the hole for the dynamic locking screw, caused by distal jamming of the tip of the nail against the anterior cortex [24]. A surgical failure due to distal jamming had never been described in the literature before [24]. Liu et al. in a retrospective review of 341 intertrochanteric fractures treated with the TFN found two patients whose nails had failed at the junction of the helical blade and the nail. Implant breakage seemed attributable to delayed union or nonunion, resulting in persistent loading of the nail and eventual fatigue of the metal. The point of insertion of the proximal blade to the nail has the narrowest cross-sectional diameter and is responsible for force transmission from the blade to the nail, explaining the propensity for fatigue fracture at this point [25]. Johnson et al. reported on 221 cases of 31 AO fractures with 22 nail breakages: 20 were Intramedullary Hip Screw® (IMHS) (Smith & Nephew™, Tennessee, USA) and 2 were Affixus® (AFF) (Zimmer Biomet™, Warsaw, Indiana, USA). All nails of this series were broken through the hole for the lag screw [26]. Therefore, there is no cephalomedullary nail reported in the literature not presenting the complication of nail breakage [13–16]. By experience [8] and literature [1–27], we know that the hole for the lag screw seems to be the weakest point, as it has a relatively small cross-sectional diameter [28]. This is the critical zone where forces from the femoral neck are transmitted to the nail in the femoral shaft [20, 29]. It has been reported that inappropriate drilling of the nail at this site due to an improperly placed drill guide, or off-center introduction of the lag screw, may damage the nail and contribute to nail breakage [30]. Salvage of failed trochanteric fracture fixation is achieved by internal fixation or arthroplasty or hemiarthroplasty [31]. The choice of the salvage procedure should consider several factors including the anatomical site of the nonunion, the quality of the remaining bone and articular cartilage, and patient factors such as age and activity level [8]. In younger patients with a well-preserved hip joint, treatment typically involves revision internal fixation with or without osteotomy or bone grafting [8]. In older patients, however, arthroplasty is indicated to help restore function and relieve pain when there is poor bone stock or a badly damaged hip joint [31, 32]. Nevertheless, total hip arthroplasty or hemiarthroplasty usually requires management of the discontinuous greater trochanter [31]. Other factors such as broken hardware, deformity, and femoral bone defects also need to be considered [31]. The use of free metal cable cerclage or dedicated plates and cerclage is to reduce the femoral open-book fracture during the femoral stem implantation [31–33]. This procedure allows earlier mobilization in older patients compared with revision internal fixation [31–33]. In our patient, we performed THA because she had an excellent femoral bone stock, osteoarthritis of the hip, good muscular trophism, and a low risk of postoperative joint dislocation. We have also met the patient's functional requests. This paper reports the first case of cephalomedullary nail breakage in two points, the holes for the lag screw and the locking screw, related to nonunion of a subtrochanteric fracture and metal fatigue in a very active elderly female.

## Figures and Tables

**Table 1:** Meta-analyses of other nail breakages compared with our experience.

Author	Total cases	Cases of broken nails	Nail type	Breakage site	Time in months	Cause of breakage
Valverde et al. [13]	223	1 (0.4%)	1st GN	Proximal	N/A	N/A
Boriani et al. [14]	1181	5 (0.4%)	1st GN	N/A	N/A	N/A
Gaebler et al. [15]	839	2 (0.2%)	1st GN	Distal	4	Direct trauma
			1st GN	Distal	5	Nonunion
Docquier et al. [16]	439	1 (0.2%)	1st or 2nd GN	N/A	N/A	Delayed union
Iwakura et al. [10]	N/A	N/A	Short 3GN	Proximal	14	Insufficient reduction
Pervez and Parker [17]	35	2 (5.7%)	Long GN	Middle	3	Delayed union
			Long GN	N/A	5	Delayed union (PF)
Van Doorn and Stapert [18]	101	2 (2.0%)	Long GN	Proximal	7	Nonunion (PF)
			Long GN	Middle	9	Nonunion (PF)
Sehat et al. [19]	100	1 (1.0%)	Long GN	Middle	N/A	Insufficient reduction
			1st GN	Proximal	7	Nonunion
			1st GN	Distal	7	Nonunion
Alvarez et al. [20]	843	5 (0.6%)	2nd GN	Proximal	7	Nonunion
			Long GN	Middle	10	Nonunion
			Long GN	Proximal	8	Nonunion
Rappold et al. [21]	61	2 (3.28%)	PFN	Proximal	12	Nonunion
			PFN	Proximal	24	Insufficient reduction
Erez and Dougherty [23]	100	1 (1%)	TN	Proximal	6	Second fall
Maniscalco et al. [24]	N/A	N/A	EN	Proximal	6	Nonunion, distal jamming
Liu et al. [25]	341	2 (0.59%)	TFN	Proximal	N/A	Nonunion, fatigue
			TFN	Proximal	NA	
Johnson et al. [26]	221	20 (9.05%)	All HIS	All proximal	Range 25–23	Nonunion, fatigue
		2 (0.90%)	AFF	All proximal		
Rollo et al. (PD)	242	3 (1.24%)	1GN	All proximal	Range 6–12	Nonunion, fatigue
Rollo et al. (PD)	286	4 (1.3%)	2GN	All proximal	Range 6–12	Nonunion, fatigue
Rollo et al. (PD)	346	1 (0.29%)	3GN (180 mm)	All proximal	Range 4–16	Nonunion, fatigue
Rollo et al. (PD)	189	2 (1.1%)	3GN (200 mm)	All proximal	Range 3–18	Nonunion, fatigue
Rollo et al. (PD)	138	0	Long GN	All proximal	Range 6–15	Nonunion, fatigue
Rollo et al. (PD)	150	2 (1.3%)	AFF	All proximal	Range 6–15	Nonunion, fatigue

1st GN: the first-generation Gamma nail; 2nd GN: the second-generation Gamma nail; 3rd GN: the third-generation Gamma nail; PFN: Proximal Femoral Nail® (DePuy Synthes, Raynham, Massachusetts, USA); TN: InterTAN® (Smith & Nephew™, Memphis, Tennessee, USA); EN: EndoViS® (Citieffe™, Calderara di Reno, Bologna, Italy); IMHS: Intramedullary Hip Screw® (Smith & Nephew™, Tennessee, USA); AFF: Affixus® (Zimmer Biomet™, Warsaw, Indiana, USA); long: long nail; proximal: the opening for the lag screw; middle: nail midshaft; distal: the opening for the distal locking screw; N/A: not available in the literature; PD: personal database; PF: pathological fracture.
